# Effects of Plant-Based Diets on Anthropometric and Cardiometabolic Markers in Adults: An Umbrella Review

**DOI:** 10.3390/nu15102331

**Published:** 2023-05-16

**Authors:** Han Shi Jocelyn Chew, Felicia Kai Xin Heng, Si Ai Tien, Jie Yun Thian, Hui Shan Chou, Shaun Seh Ern Loong, Wei How Darryl Ang, Nicholas W. S. Chew, Ka-Hei Kenneth Lo

**Affiliations:** 1 Alice Lee Centre for Nursing Studies, Yong Loo Lin School of Medicine, National University of Singapore, Singapore 117597, Singapore; 2 Department of Nursing, Ng Teng Fong General Hospital, Singapore 609606, Singapore; 3 Department of Nursing, Khoo Teck Puat Hospital, Singapore 768828, Singapore; 4 Department of Nursing, Tan Tock Seng Hospital, Singapore 308433, Singapore; 5 Department of Nursing, Singapore General Hospital, Singapore 169608, Singapore; 6 Yong Loo Lin School of Medicine, National University of Singapore, Singapore 117597, Singapore; 7 Department of Cardiology, National University Heart Centre, Singapore 119074, Singapore; 8 Department of Food Science and Nutrition, The Hong Kong Polytechnic University, Hong Kong

**Keywords:** plant-based, diet, nutrition, body weight gain, cardiometabolic, blood glucose

## Abstract

We conducted an umbrella review to consolidate the evidence of adopting plant-based diets on anthropometric and cardiometabolic outcomes. Six electronic databases (CINAHL, EMBASE, PubMed, Scopus, the Cochrane Library, and Web of Science) were searched for systematic reviews with meta-analysis (SRMAs) published from each journal’s inception until 1 October 2022. Effect sizes from SRMAs and primary studies were pooled separately using random effects models. Overlapping primary studies were removed for primary studies’ analyses. Seven SRMAs representing 51 primary studies were included, suggesting significant benefits of plant-based diets on weight (−2.09 kg, 95% CI: −3.56, −0.62 kg, *p* = 0.01, *I*^2^ = 95.6%), body mass index (−0.95 kg/m^2^, 95% CI: −1.26, −0.63 kg/m^2^, *p* = 0.002; *I*^2^ = 45.1%), waist circumference (−2.20 cm, 95% CI: −0.08, 0.00 cm, *p* = 0.04; *I*^2^ = 88.4%), fasting blood glucose (−0.11 mmol/L, 95% CI: −0.13, −0.09 mmol/L, *p* < 0.001, *I*^2^ = 18.2%), and low-density lipoprotein cholesterol (−0.31 mmol/L, 95% CI: −0.41, −0.20 mmol/L, *p* < 0.001, *I*^2^ = 65.6%). Changes in high-density lipoprotein cholesterol, triglycerides, and blood pressure were not statistically significant. Generally, plant-based diets were recommended to improve anthropometry, lipid profile, and glucose metabolism. However, findings should be interpreted with caution, because most of the reviews were rated to be of low credibility of evidence and were largely based on Western eating habits and traditions, which may limit the generalizability of findings.

## 1. Introduction

There is a fine line between plant-based and vegetarian diets in that the former allows a limited consumption of animal-based products, whereas the various types of vegetarian diets strictly limit the consumption of specific animal-based products. Plant-based diets have been gaining popularity for their anthropometric and cardiometabolic benefits. Studies have shown that adopting a plant-based diet results in up to 3.9 kg of weight loss in adults with obesity or overweight [1,2]. Adopting a plant-based diet has also been shown to reduce cardiovascular and all-cause mortality by up to 32% and 25%, respectively [3]. Such benefits have been associated with the lower amounts of calories and saturated fats that are present in plant-based foods (versus animal-based foods) [4], with higher amounts of phytochemicals (e.g., polyphenols, flavonoids, and alkaloids) and bioactive substances (e.g., coenzyme Q10 and plant sterol) [5], and with improving gut microbiota [6]. Such effects have been linked to the suppression of lipid accumulation and adipogenesis, reducing the risk of hyperlipidemia and obesity [7]. Plant-based diets, such as low-fat vegan diets, have also been found to improve the balance in gut microbiota [6], for example, by increasing *Faecalibacterium prausnitzii* and decreasing *Bacteroides fragilis*, which reduces body weight, fat mass, and visceral fat and creates an increase in insulin sensitivity [8]. A balanced gut microbiota has also been linked to the reduction in hepatic trimethylamine-N-oxide (TMAO), reducing the risk of cardiovascular diseases by reducing inflammation and endothelial cell dysfunction [9].

According to a recent article that aimed to clarify the definitions, a vegetarian diet is defined as “a dietary pattern that excludes meat, meat-derived foods, and, to different extents, other animal products” [10]. A plant-based diet is defined as “a dietary pattern in which foods of animal origin are totally or mostly excluded” [10]. Vegetarian and vegan diets are a category of plant-based diets that restrict animal products such as meat, poultry, or fish [3]. Several umbrella reviews have examined the effects of vegetarian and vegan diets but not plant-based diets. An umbrella review reported that adopting a vegetarian diet significantly lowered the risk of coronary heart disease by close to 30% [11]. However, this was based on only one SRMA, which included eight primary studies on the risk of cardiovascular mortality, instead of examining the change in cardiometabolic markers [12]. Another umbrella review reported that adopting a vegetarian diet resulted in significant weight loss in people with type 2 diabetes mellitus (T2DM), but it only included one SRMA that focused specifically on dietary pulses and legumes [13,14]. Another umbrella review reported the positive health effects of adopting a vegetarian diet on cholesterol and body weight, but included studies of various designs (e.g., cross-sectional, case-control, cohort, and clinical trials) instead of just randomized controlled trials, which are of the highest quality of evidence [15]. A vegan diet was also found to reduce body weight significantly and lower the risk of all-cause mortality, but the meta-analysis results were pooled without accounting for the potential overlaps in primary study findings, therefore potentially overestimating the sample and effect sizes [16].

Due to the various methodological limitations in the existing relevant umbrella reviews, we conducted an umbrella review to consolidate the vast amount of evidence from existing SRMAs on the effects of plant-based diets on anthropometric and cardiometabolic markers, while considering the magnitude, precision, and potential bias in the existing evidence [17].

## 2. Materials and Methods

This umbrella review was reported according to the preferred reporting items for systematic reviews and meta-analyses (PRISMA) guidelines (Supplementary S1) and registered with PROSPERO (ref: CRD42023393048) [18].

### 2.1. Search Strategy

A preliminary search was first conducted on PubMed, Google Scholar, and Prospero to avoid duplicating any existing umbrella reviews on this topic. Six electronic databases (i.e., CINAHL, EMBASE, PubMed, Scopus, the Cochrane Library, and Web of Science) were searched for articles published from journal inception to 1 October 2022. Keywords were derived from an initial search of articles on PubMed and the medical subject heading terms. Search terms included plant-based diet, plant-based nutrition, plant-based food, vegetarian, vegan, lacto-vegetarian, lacto-ovo vegetarian, vegan, and plant foods (Supplementary S2: search strategies used for each database).

### 2.2. Study Selection

Citations were managed using the EndNote X20 software. Studies were included if they included: (1) adults who were healthy or had cardiometabolic diseases; (2) plant-based diets defined as the consumption of mostly plant-based and not animal foods, including vegan, vegetarian, lacto-vegetarian, lacto-ovo-vegetarian, pesco-vegetarian, and semi-vegetarian diets [19]; (3) comparisons with non-plant-based diets; (4) body weight as the primary outcome; (5) systematic reviews with meta-analysis of data from experimental studies; (6) internationally refereed journal articles; (7) articles written in English. Articles were excluded if they included: (1) adults with pregnancy, adults with diseases other than cardiometabolic diseases, or people less than 18 years old; (2) disease-specific nutrition therapy and specific plant-based food items instead of a plant-based dietary pattern; (3) other forms of review such as scoping reviews, integrative reviews, critical reviews, narrative reviews, and systematic reviews without meta-analysis. Titles, abstracts, and full texts were screened independently by three reviewers (FKXH, SAT, and JYT) and discrepancies were resolved by a fourth reviewer (HSJC). Cohen’s kappa (*k*) statistic was used to assess the interrater agreements for study selection, methodological quality appraisal, and level of evidence assessment. Agreements were assessed according to the following: no agreement (*k* ≤ 0), none to slight (*k* = 0.01–0.20), fair (*k* = 0.21–0.40), moderate (*k* = 0.41–0.60), substantial (*k* = 0.61–0.80), and almost perfect agreement (*k* = 0.81–1.00) [20].

### 2.3. Data Extraction

Three reviewers (FKXH, SAT, and JYT) completed the data extraction independently using an a priori data extraction Excel file; the data extracted were validated by a fourth reviewer (HSJC). Information retrieved was: first author, year, type of plant-based diet, study design, number of randomized controlled trials (RCTs) or non-randomized studies of intervention (NRSI), number of databases, search period, publication year, countries, sample size, population characteristics, mean age, mean BMI, outcomes, duration of the diet, methodological quality assessment tool, certainty of the evidence assessment tool, protocol number, type of analyses, and a list of primary studies included in each meta-analysis. Central tendencies and variance estimates of each outcome were also retrieved from the systematic reviews and primary articles included in each systematic review.

### 2.4. Methodological Quality Appraisal

Assessing the methodological quality of systematic review 2 (AMSTAR 2) was used to assess the methodological quality of the included SRMAs. The AMSTAR 2 comprised 7 critical (i.e., the prospective registration of protocol, search strategy, justifications for exclusion of studies, quality assessment of included studies, appropriateness of analysis method, consideration of quality when interpreting results, and presence of publication biases) and 9 non-critical domains. The domains were rated either yes, partially yes, or no and rated overall to be high (only ≤1 item in a non-critical domain rated as yes), moderate (>1 item in a non-critical domain rated as yes), low (1 item in a critical domain rated as yes regardless of ratings in the non-critical domain), or critically low (>1 item in a critical domain rated as yes regardless of whether rated in non-critical domains) quality [21].

### 2.5. Certainty of Evidence

Each meta-analysis’ level of evidence was classified into one of four classifications, namely: Class I, where the number of cases was >1000, the *p*-value was <10^−6^, *I*^2^ < 50%, the 95% prediction interval excludes the null, there were no small-study effects, and there were no other significant biases; Class II, where the number of cases was >1000, the *p*-value was <10^−6^, the largest study had a statistically significant effect, and the Class I criteria were not met; Class III, where the number of cases was >1000, the *p*-value was <10^−3^, and the Class I and II criteria were not met; Class IV, where the *p*-value was <0.05 and the Class I–III criteria were not met and non-significant when only the *p*-value was >0.05 [22].

### 2.6. Data Analyses

Effect sizes from the included SRMAs and their respective primary studies were pooled using the generic inverse variance, where larger studies with lower standard errors were given more weight to enhance precision. Pooled effect sizes were presented as weighted mean difference (WMD) and 95% confidence interval (95% CI). All meta-analyses were performed using a Hartung–Knapp–Sidik–Jonkman (HKSJ) estimated random effects model and the overall effect sizes were estimated alongside prediction intervals [23]. Overlapping studies were removed from the meta-analyses to avoid overestimating the treatment effects [24]. For studies that included more than two arms, only the interventional arm that included a plant-based diet was used in comparison with the control arm with a non-plant-based diet. Between-study heterogeneity was assessed using the Cochran *Q* statistics and quantified using the *I*^2^ statistics (>50% indicates heterogeneity). Subgroup analyses were performed for different types of plant-based diets (i.e., plant-based diets including vegan, vegetarian, lacto-vegetarian, lacto-ovo-vegetarian, and non-specific plant-based diets) when 10 or more studies reported on a specific outcome. Publication bias was assessed using funnel plots and Egger’s test (*p* < 0.01 indicates a small-study effect). Cardiometabolic markers measured in mg/dL were converted to mmol/L. Sensitivity analyses were performed using the leave-one-out method. The extent of overlapping studies was represented by the corrected covered area (CCA) [25]:

$$\mathrm{CCA}=\frac{N-r}{\mathrm{rc}-r}$$

where N represents the total number of included publications in the reviews, r represents the number of primary studies, and c represents the number of included reviews [25]. The CCA score is interpreted as having a slight overlap (0–5%), moderate overlap (6–10%), high overlap (11–15%), and very high overlap (>15%) [25]. All analyses were performed using R version 4.1.3 [26] with packages meta [27] and metafor [28].

## 3. Results

**Figure 1:**
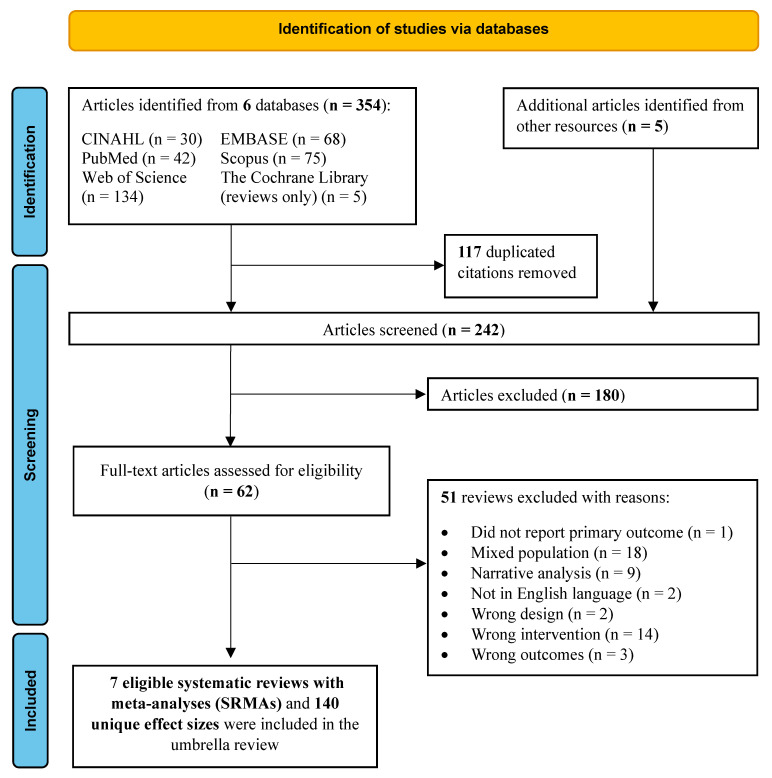
PRISMA 2020 flow diagram of review selection for umbrella review.

A total of 354 citations were initially retrieved from the database search (Figure 1). After removing 117 duplicated articles, 242 titles and abstracts were screened, of which 180 citations were removed. After the full-text screening of 62 articles, 7 SRMA [29,30,31,32,33,34,35] and 140 unique effect sizes were analyzed in this umbrella review. The Cohen’s *k* for article selection and AMSTAR 2 rating were 0.78 and 0.77, respectively, indicating substantial agreement.

### 3.1. Study Characteristics

A summary of the study characteristics is shown in Table 1. The seven studies were published between 2015 and 2022 and represented 51 unique primary studies, with 4569 participants aged 18 to 81 and sample sizes ranging from 269 [29] to 1511 [32]. The primary studies included were published from 1947 to 2021 and were searched from journal inceptions up until March 2022. The types of plant-based diets included semi-vegetarian, pesco-vegetarian, lacto-vegetarian, lacto-ovo vegetarian, vegan, and Nordic diets. Plant-based diets were compared with usual diets, of which five articles [29,30,31,32,34] reported the duration of plant-based diets ranging from 6 weeks to more than 2 years. One study did not report the use of a methodological quality assessment tool [35] and only three studies reported the use of the certainty of evidence assessment tool (GRADE) [33,34,35]. All seven studies used meta-analyses with random effects models, of which effect sizes are shown in Table 2. The credibility of evidence was rated as weak (Class IV) for all pooled effect sizes, except for 1, which was rated as convincing (Class I) [30].

### 3.2. Quality Assessment

The methodological qualities of the SRMAs were limited by several factors (Table S1). Regarding the critical domains, most of the SRMAs (n = 5) were downgraded, as the study reviewers did not provide a list of excluded studies or justify the exclusions. In addition, two reviews did not adopt appropriate quality assessments for their included studies. Finally, one review did not assess the potential impact of a risk of bias on the review findings. For these reasons, the methodological qualities of two SRMAs were rated as low [31,32], four were rated as moderate [29,30,33,34], and one was rated as high [35].

### 3.3. Anthropometric Outcomes

**Figure 2:**
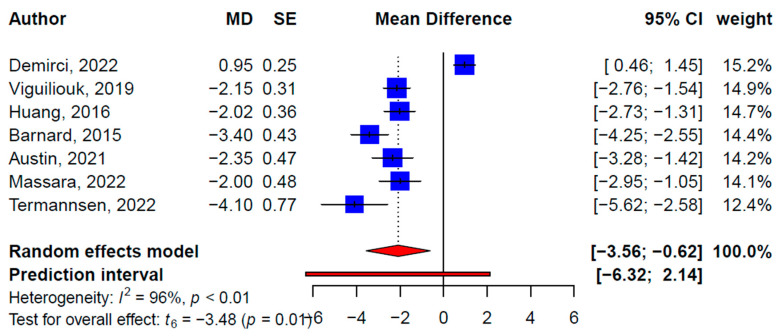
Pooled effect size of plant-based diets on weight (kg) based on findings from the included systematic reviews with meta-analysis [29,30,31,32,33,34,35].

**Figure 3:**
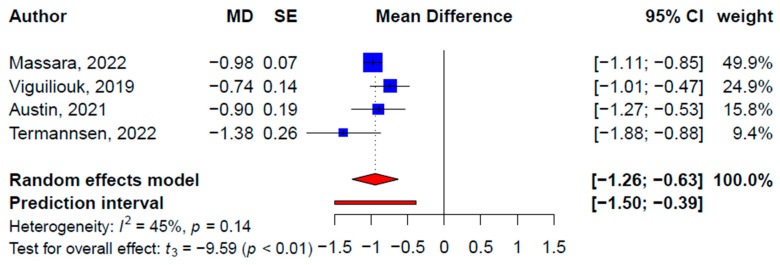
Pooled effect size of plant-based diets on body mass index (kg/m^2^) based on findings from the included systematic reviews with meta-analysis [29,33,34,35].

**Figure 4:**
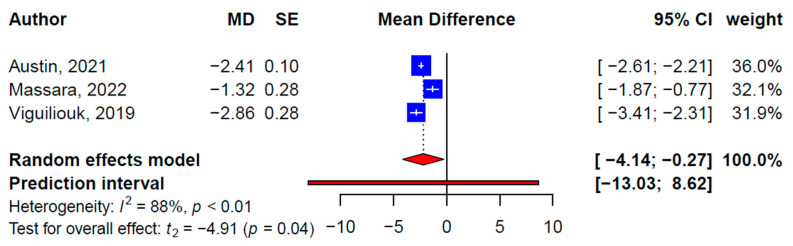
Pooled effect size of plant-based diets on waist circumference (cm) based on findings from the included systematic reviews with meta-analysis [29,33,35].

Seven, four, and three SRMAs reported statistically significant effects of plant-based diets on weight (−2.09 kg, 95% CI: −3.56, −0.62 kg, *p* = 0.01, *I*^2^ = 95.6%) (Figure 2), BMI (−0.95 kg/m^2^, 95% CI: −1.26, −0.63 kg/m^2^, *p* = 0.002; *I*^2^ = 45.1%) (Figure 3), and waist circumference (−2.20 cm, 95% CI: −0.08, 0.00 cm, *p* = 0.04; *I*^2^ = 88.4%) (Figure 4), respectively. Meta-analyses of the primary studies produced similar findings based on 35, 26, and 14 primary studies’ effect sizes, respectively (Table 3, Figures S1–S3). For instance, the pooled effect size from the primary studies on weight loss, excluding overlapping studies, was 2.90 kg (95% CI: −3.62, −2.18 kg, *p <* 0.001; *I*^2^ = 82.6%). The subgroup analysis did not show statistically significant moderating effects of the different types of plant-based diets on weight, BMI, or waist circumference. No publication bias was detected on the primary study levels; the CCA for weight, BMI, and waist circumference were 6.5%, 7.2%, and 5.9%, respectively.

**Figure 5:**
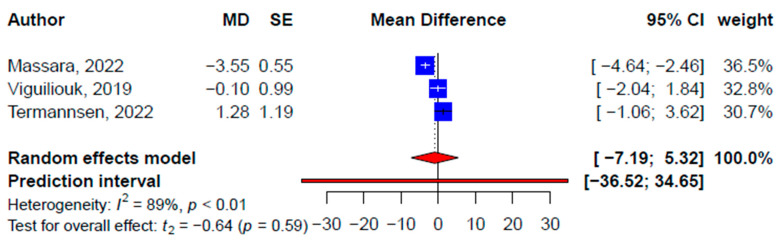
Pooled effect size of plant-based diets on systolic blood pressure (mmHg) based on findings from the included systematic reviews with meta-analysis [33,34,35].

**Figure 6:**
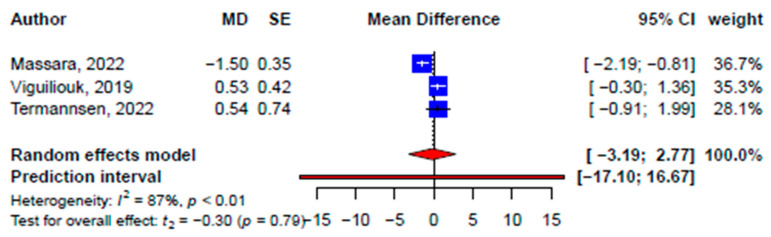
Pooled effect size of plant-based diets on diastolic blood pressure (mmHg) based on findings from the included systematic reviews with meta-analysis [33,34,35].

**Figure 7:**
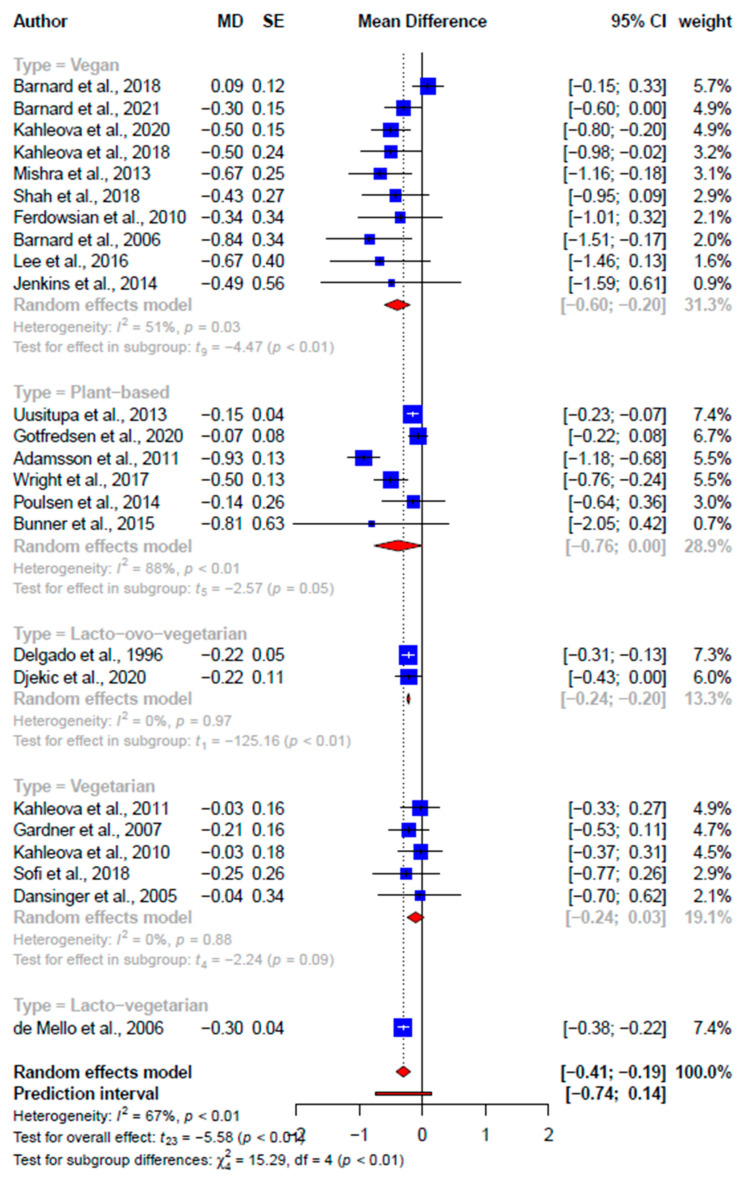
Forest plot comparing the different primary studies’ effect sizes between different plant-based diets on low-density lipoprotein (mmol/L) [2,36,37,38,39,40,41,42,43,44,45,46,47,48,49,50,51,52,53,54,55,56,57].

**Figure 8:**
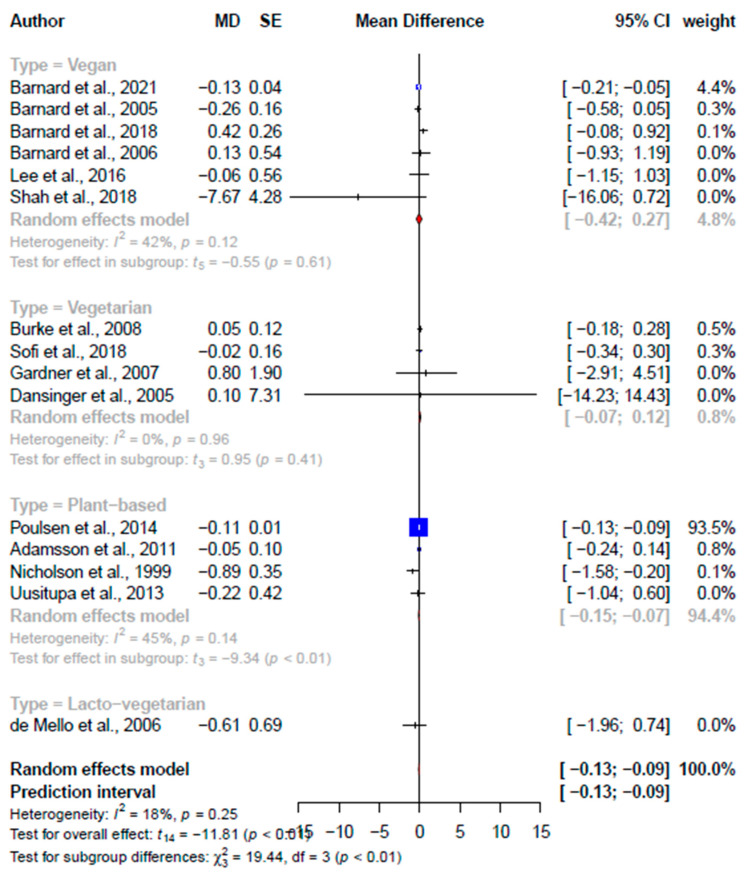
Forest plot comparing the different primary studies’ effect sizes between different plant-based diets on fasting blood glucose (mmol/L) [30,37,38,41,43,44,46,48,49,54,55,56,57,58,59].

Findings from the meta-analyses of SRMAs and primary studies’ data were consistent with the improvements in high-density lipoprotein cholesterol (HDL-C) (Figure S4) and systolic (Figure 5) and diastolic blood pressure (Figure 6) but not (Figure S4) low-density lipoprotein cholesterol (LDL-C) (Figure S5) or triglyceride (Figure S6; Table 3). While the improvement in LDL-C was not statistically significant at the pooled SRMA level (−0.18 mmol/L, 95% CI: −0.38, 0.01 mmol/L, *p* = 0.06, *I*^2^ = 49.7%), significant improvement was found at the primary study level (−0.30 mmol/L, 95% CI: −0.41, −0.19 mmol/L, *p* < 0.001, *I*^2^ = 66.5%) (Figure 7). Similarly, pooled effects on triglycerides at both the SRMA (0.04 mmol/L, 95% CI: −0.22, 0.31 mmol/L, *p* = 0.55, *I*^2^ = 66.3%) and primary study (0.39 mmol/L, 95% CI: −0.06, 0.85 mmol/L, *p* = 0.09, *I*^2^ = 91.4%) (Figure S7) levels were not statistically significant (Figure 8). Subgroup differences were found for the difference in plant-based diets on the pooled effects on LDL-C, triglyceride, and systolic and diastolic blood pressure. Publication bias was detected in the meta-analysis of primary studies on systolic blood pressure (2.64, *p* = 0.001) (Figure S8) and diastolic blood pressure (2.26, *p* = 0.002) (Figure S9). The SRMAs included for both lipid profile and blood pressure were the same and the CCA was 2.17%.

**Table 3:** Meta-analysis results of the effect of plant-based diets on weight, BMI, waist circumference, lipid profile, glucose metabolism profile, and blood pressure from the meta-analysis and study level data.

Outcomes and Subgroups	Meta-Analysis Level					Study Level					
	Number of Reviews	MD (95% CI)	*t*	*p*-Value	*I*^2^, %	Number of Study Arms	MD (95% CI)	*t*	*p*-Value	*I*^2^, %	Subgroup Differences *Q*, *p*-Value
**Weight, kg**											
Total	7	−2.09 (−3.56, −0.62)	−3.48	* 0.01	95.6	35	−2.90 (−3.62, −2.18)	−8.16	*** <0.001	89.4	
Subgroups											4.63, 0.33
Vegan	-	-	-	-	-	13	−3.25 (−4.34, −2.17)	−6.53	<0.01	87.4	-
Plant-based	-	-	-	-	-	9	−3.54 (−5.89, −1.18)	−3.47	<0.01	94.3	-
Vegetarian	-	-	-	-	-	7	−1.95 (−3.15, −0.76)	−4.00	<0.01	83.1	-
Lacto-ovo-vegetarian	-	-	-	-	-	5	−2.48 (−6.05, 1.09)	−1.93	0.13	68.7	-
Lacto-vegetarian	-	-	-	-	-	1	−3.20 (−4.77, −1.63)	-	-	-	-
**Body mass index, kg/m^2^**											
Total	4	−0.95 (−1.26, −0.63)	−9.59	** 0.002	45.1	26	−0.82 (−1.28, −0.37)	−3.71	** 0.001	82.6	
Subgroups	-	-	-	-	-						3.79, 0.15
Vegan	-	-	-	-	-	11	−0.80 (−1.67, 0.06)	−2.07	0.06	86.3	-
Plant-based	-	-	-	-	-	5	−1.65 (−3.18, −0.11)	−2.98	0.04	73.3	-
Vegetarian	-	-	-	-	-	7	−0.55 (−1.04, −0.06)	−2.77	0.03	78.5	-
Lacto-ovo-vegetarian	-	-	-	-	-	2	−1.63 (−22.43, 19.15)	−1.00	0.5	77.6	-
Lacto-vegetarian	-	-	-	-	-	1	0.38 (−0.72, 1.48)	-	-	-	-
**Waist circumference, cm**											
Total	3	−2.20 (−4.14, −0.27)	−4.91	* 0.04	88.4	14	−2.16 (−4.07, −0.25)	−2.45	* 0.03	92.9	-
Subgroups	-	-	-	-	-						1.79, 0.62
Vegan	-	-	-	-	-	4	−1.75 (−4.88, 1.38)	−1.55	0.2	89.4	-
Plant-based	-	-	-	-	-	5	−4.32 (−12.56, 3.91)	−1.67	0.19	95.2	-
Vegetarian	-	-	-	-	-	4	−1.16 (−3.50, 1.18)	−1.58	0.21	64.9	-
Lacto-vegetarian	-	-	-	-	-	1	−0.50 (−3.67, 2.67)	-	-	-	-
**High-density lipoprotein cholesterol (HDL-C), mmol/L**											
Total	3	−0.04 (−0.08, 0.00)	−4.11	0.05	0	30	−0.03 (−0.15, 0.08)	−0.55	0.59	94.2	
Subgroups	-	-	-	-	-						5.95, 0.20
Vegan	-	-	-	-	-	12	0.05 (−0.24, 0.34)	0.37	0.72	97.1	-
Plant-based	-	-	-	-	-	7	−0.06 (−0.19, 0.08)	−1.05	0.33	90.7	-
Vegetarian	-	-	-	-	-	7	−0.07 (−0.11, −0.03)	−4.16	<0.01	6.2	-
Lacto-ovo-vegetarian						3	−0.16 (−0.82, 0.50)	−1.05	0.04	78.7	-
Lacto-vegetarian	-	-	-	-	-	1	−0.01 (−0.05, 0.03)	-	-	-	-
**Low-density lipoprotein cholesterol (LDL-C) ^a^, mmol/L**											
Total	3	−0.18 (−0.38, 0.01)	−4.00	0.06	49.7	24	−0.30 (−0.41, −0.19)	−5.58	*** <0.001	66.5	
Subgroups	-	-	-	-	-						^†^ 15.29, 0.004
Vegan	-	-	-	-	-	10	−0.40 (−0.60, −0.20)	−4.47	<0.01	50.7	-
Non-specific	-	-	-	-	-	6	−0.38 (−0.76, −0.00)	−2.57	0.05	88.2	-
Vegetarian	-	-	-	-	-	5	−0.11 (−0.24, 0.03)	−2.24	0.09	0	-
Lacto-ovo-vegetarian	-	-	-	-	-	2	−0.22 (−0.24, −0.20)	−125.16	<0.01	0	-
Lacto-vegetarian	-	-	-	-	-	1	−0.30 (−0.38, −0.22)	-	-	-	-
**Triglyceride, mmol/L**											
Total	3	0.04 (−0.22, 0.31)	0.71	0.55	66.3	26	0.39 (−0.06, 0.85)	1.77	0.09	91.4	
Subgroups											^†^ 11.9, 0.04
Vegan	-	-	-	-	-	11	0.82 (−0.17, 1.81)	1.84	0.10	95.5	-
Plant-based	-	-	-	-	-	6	0.09 (−0.12, 0.30)	1.12	0.31	52.6	-
Vegetarian	-	-	-	-	-	5	−0.18 (−0.41, 0.05)	−2.16	0.10	19.7	-
Lacto-ovo-vegetarian						3	0.00 (−0.20, 0.20)	0.02	0.98	0.0	-
Lacto-vegetarian	-	-	-	-	-	1	0.86 (−0.18, 1.90)	-	-	0.0	-
**Systolic blood pressure, mmHg**											
Total	3	−0.93 (−7.19, 5.32)	−0.64	0.59	89.4	21	0.07 (−1.97, 2.10)	0.07	0.95	91.9	-
Subgroups	-	-	-	-	-						^†^ 4.41, 0.04
Vegan	-	-	-	-	-	9	1.56 (−2.41, 5.53)	0.91	0.39	83.9	-
Plant-based	-	-	-	-	-	8	−1.48 (−5.10, 2.14)	−0.97	0.36	91.4	-
Vegetarian	-	-	-	-	-	2	0.96 (−10.50, 12.43)	1.07	0.48	0	-
Lacto-ovo-vegetarian	-	-	-	-	-	2	−1.51 (−8.76, 5.74)	−2.64	0.23	0	-
**Diastolic blood pressure, mmHg**											
Total	3	−0.21 (−3.19, 2.77)	−0.3	0.79	87.4	21	0.01 (−1.41, 1.43)	0.02	0.99	88.9	-
Subgroups	-	-	-	-	-					-	^†^ 131.2, <0.001
Vegan	-	-	-	-	-	9	0.61 (−2.10, 3.31)	0.52	0.62	82.0	-
Plant-based	-	-	-	-	-	8	−0.73 (−3.62, 2.16)	−0.60	0.57	89.1	-
Vegetarian	-	-	-	-	-	2	1.16 (−1.34, 3.66)	5.92	0.11	0	-
Lacto-ovo-vegetarian	-	-	-	-	-	2	−1.35 (−2.78, 0.08)	−12.02	0.05	0	-
**Fasting blood glucose, mmol/L**											
Total	3	−0.06 (−0.33, 0.21)	−0.97	0.44	84.0	15	−0.11 (−0.13, −0.09)	−11.8	*** <0.001	18.2	
Subgroups	-	-	-	-	-						^†^ 26.9, <0.001
Vegan	-	-	-	-	-	6	−0.07 (−0.42, 0.27)	−0.55	0.61	42.1	-
Plant-based	-	-	-	-	-	4	−0.11 (−0.15, −0.07)	−9.34	<0.01	44.8	-
Vegetarian	-	-	-	-	-	4	0.03 (−1.97, 0.75)	0.95	0.41	0.0	-
Lacto-vegetarian	-	-	-	-	-	1	−0.61 (−1.97, 0.95)	-	-	-	-
**HbA1c ^b^**											
Total	-	-	-	-	-	13	−0.03 (−0.06, 0.00)	−1.83	0.09	32.4	-
Subgroups	-	-	-	-	-						^†^ 43.3, <0.001
Vegan	-	-	-	-	-	9	−0.06 (−0.08, −0.04)	−5.83	<0.01	0.0	-
Plant-based	-	-	-	-	-	2	0.01 (0.00, 0.01)	22.13	0.03	0.0	-
Vegetarian	-	-	-	-	-	1	−0.44 (−0.97, 0.09)	-	-	-	-
Lacto-ovo-vegetarian	-	-	-	-	-	1	−0.00 (−0.02, 0.02)	-	-	-	-

MD—mean difference; CI—confidence interval; HbA1c—hemoglobin A1c; *t—t*-statistic; ^†^ indicates a statistically significant subgroup effect; ^a^ removed studies by Burke et al., 2008 [58], Kahleova et al., 2013 [60], and Mahon et al., 2007 [61] based on leave-out-one sensitivity analysis; ^b^ removed study by Kahleova et al., 2020 [36] based on leave-out-one sensitivity analysis; *p* < 0.1 for *Q* statistic; *** *p* < 0.001; ** *p* < 0.01; * *p* < 0.05. Hartung–Knapp–Sidik–Jonkman (HKSJ) method was used for random effects meta-analysis. Plant-based refers to non-specific plant-based diets.

#### Glucose Metabolism

Significant improvements were found in the pooled effect on fasting blood glucose (−0.11 mmol/L, 95% CI: −0.13, −0.09 mmol/L, *p* < 0.001, *I*^2^ = 18.2%) (Figure 8) but not on HbA1c (−0.03 mmol/L, 95% CI: −0.06, 0.00, *p* = 0.09, *I*^2^ = 32.4%) (Figure S10) at the primary study level. At the SRMA level, effect sizes were only pooled for fasting blood glucose, which showed no significant improvements (−0.06 mmol/L, 95% CI: −0.33, 0.21, *p* = 0.44, *I*^2^ =84.0%) (Figure S11), and not for HbA1c, as only two studies were available. There was no overlap in the articles on glucose metabolism.

## 4. Discussion

Through this umbrella review, we found significant effects (consistent between pooled meta-analysis and primary study effect sizes) of various plant-based diets on body weight (−2.90 kg, 95% CI: −3.62; −2.18 kg), BMI (−0.82 kg/m^2^, 95% CI: −1.28; −0.37 kg/m^2^), and waist circumference (−2.16 cm, 95% CI: −4.07; −0.25 cm) but not systolic blood pressure (0.07 mmHg, 95% CI: −1.97; 2.10 mmHg), diastolic blood pressure (0.01 mmHg, 95% CI: −1.41; 1.43 mmHg), HDL-C (−0.03 mmol/L, 95% CI: −0.15; −0.08 mmol/L), or triglyceride (0.39 mmol/L, 95% CI: −0.06; −0.85 mmol/L). The pooled effect sizes from the primary studies, excluding overlapping studies, indicated significant improvements in LDL-C (−0.31 mmol/L, 95% CI: −0.41; −0.20 mmol/L) and fasting blood glucose (−0.11 mmol/L, 95% CI: −0.13; −0.09 mmol/L). However, the results from the pooled SRMA effect sizes were not significant, potentially due to the difference in meta-analytic weight assigned based on the number of primary studies included instead of the sample size. Results should also be interpreted cautiously, as all except one SRMA effect size was rated as having weak certainties of evidence. This is similar to an umbrella review that graded all of the nine effect sizes on the effects of vegetarian diets as weak [62].

While plant-based diets had positive effects on anthropometry, the non-significant negative effects on HDL-C and triglyceride were surprising but not unprecedented. In a cross-sectional study, an increase in the plant-based diet index was found to be associated with a 2.16 higher odds of having a higher triglyceride level [63]. This was attributed to the substitution of high-fat animal-based food with refined carbohydrate-rich and sugar-laden plant-based foods, which could have reduced HDL-C and increased triglycerides [63]. However, the change in specific food items consumed was rarely reported in relevant empirical studies and SRMAs, rendering it difficult to ascertain this speculation. Concurrently, two of the three SRMAs that reported an increase in triglyceride levels were observed in participants with diabetes mellitus and overweight [34,35], who may have had the habit of consuming a high-sugar diet that could have been augmented by the reduction of animal-based food consumption. Similarly, another umbrella review found that adopting a vegan diet increased triglyceride levels, potentially due to the influence on blood lipid metabolism, where a reduction in fat intake and an increase in carbohydrate intake causes an increase in triglyceride release to the bloodstream [64]. This suggests that purely adopting a plant-based diet may not be sufficient in improving blood lipid markers and that the diet modification should be complemented with a high-quality intake of carbohydrates and fats instead of consuming unhealthy plant-based foods such as potato chips. For example, one study found that substituting common refined carbohydrates such as white rice with pasta, which has a lower glycemic index and hence produces a lower post-prandial insulin spike, was associated with a lower risk of stroke and atherosclerotic cardiovascular diseases [65].

Non-significant effects on blood pressure resonated with an existing umbrella review, where the effects of plant-based diets, including vegetarian diets, on blood pressure were inconsistent [66]. This was speculated to be due to the difference in participant profiles and the inconsistency of what constitutes a specific plant-based diet (e.g., type of plant-based food item, portion, sauces, etc.) [66]. Similarly, the SRMA [33] that showed a relatively larger reduction in blood pressure included only studies conducted in Europe, while the other two included studies from more varied regions, suggesting more generalized findings [34,35]. Long-term randomized controlled trials examining the impact of a plant-based diet on various health outcomes, including blood pressure, will help to enhance the clinical relevance and effectiveness of diet-based cardiometabolic disease prevention and management. This will further refine the understanding between a plant-based diet and blood pressure. However, the causal relationship between the consumption of a plant-based diet and blood pressure remains unclear. Therefore, with inconsistent results, it is not prudent to recommend a plant-based diet to improve the level of blood pressure.

Regarding the quality assessment of the included reviews, the findings of the AMSTAR 2 also identified several lapses during the review process that led to a poorer rating. The PRISMA guideline proposed that SRMAs should include a transparent search process [18]; this could be in the form of the inclusion of a list of excluded studies with justifications. This would allow readers to comprehend the study selection process and would prevent reviewers from omitting studies that otherwise met the eligibility criteria. Further, the use of appropriate quality assessment tools would be helpful for providing clinicians insight into the quality of the evidence [18]. This would be exceptionally helpful for individuals who are not adept at statistics to make sense of the findings and would support their decision in the evidence translation process.

### Strengths and Limitations

This umbrella review has several strengths. It was carried out according to well-established systematic review guidelines. The study was prospectively registered, and an extensive search of the literature was carried out to diminish the possibility of publication bias. All the screenings and assessments were performed independently. It provided an intensive synthesis of all the currently available evidence of the potential benefits of adopting a plant-based diet in terms of the anthropometric and cardiometabolic markers. A systematic search strategy was formulated to obtain all RCTs, which further strengthened the conclusion. The inclusion of RCTs helped to reduce confounding between known and unknown sources due to the randomization. Additionally, the quality of the systematic review and meta-analyses were assessed using AMSTAR 2. It is a critical appraisal tool published in 2017 that provides a standardized approach for assessing the methodological quality of systematic reviews. It helps to establish whether the most important elements are reported. The Cohen’s *k* for article selection and AMSTAR 2 rating were 0.78 and 0.77, respectively, indicating substantial agreement.

However, this umbrella review is not devoid of limitations. Some of the included meta-analyses were evaluated to be of “low” AMSTAR 2 quality. More efforts are needed to improve the quality of published articles and to further research the effect of plant-based diets on anthropometric and cardiometabolic markers in adults to allow a conclusive conclusion to be drawn. This will help to facilitate the understanding, meaning, and applicability of findings in clinical practice. At the same time, there were several meta-analyses with less than ten RCTs to permit the assessment of publication bias via funnel plot. Moreover, the included studies were largely based on Western eating habits and traditions, which could limit the generalizability of our findings to other populations with different genetic makeups and food preferences [67].

## 5. Conclusions

The potential benefits identified in this umbrella review suggest that, in broad terms, the adoption of a plant-based diet is recommended. Therefore, it should be endorsed as a public health goal. However, it should be noted with caution that the review is largely based on Western eating habits and traditions. Eating patterns can be culturally sensitive and vary vastly in different settings. Hence, it may not be fully applicable from a global point of view. From a methodological perspective, the findings from the AMSTAR 2 also proposed that future SRMAs should adopt robust methods (e.g., prospective registration and appropriate quality assessment) and adhere to the reporting guidelines such as PRISMA.

## Figures and Tables

**Table 1:** Characteristics of included systematic reviews with meta-analyses.

Author, Year	No. of RCTs/Participant Characteristics/Age */Sample Size	Countries/Year Range	No. of Databases/Search Period	Types of Plant-Based Diet/Duration	Outcomes	Quality Assessment/Certainty of Evidence/Protocol Number	AMSTAR 2 Rating
Austin, 2021 [29]	7/participants with T2DM/57.1/269	USA, Czech Republic, Republic of Korea/1999–2018	4 (Cochrane Library, CINAHL, MEDLINE, and EMBASE)/inception until April 2021	Semi-vegetarian, pesco-vegetarian, lacto-ovo vegetarian, and vegan/6–22 weeks	Weight, BMI, waist circumference	Quality criteria checklist for primary research ^#^/NR/CRD42021222987	Moderate
Barnard, 2015 [30]	15/general adults/NR/755	Sweden, Norway, Spain, Finland, USA, Poland/1947–2013	3 (PubMed, Embase, the Cochrane Central Register of Controlled Trials)/until 31 December 2013	Vegan or vegetarian diet/4 weeks > 2 years	Weight	ROB/NR/CRD42012003506	Moderate
Demirci, 2022 [31]	11/participants with T2 DM and overweight/NR/934	USA, Sweden, Czechia, Republic of Korea, Italy, New Zealand/2007–2021	3 (PubMed, Science Direct, ResearchGate)/inception until 2021	Vegetarian diet/4–72 weeks	Weight	Jadad score/NR	Low
Huang, 2016 [32]	12/general adults/18 to 82 years/1151	NR/1950 to 22 September 2014	3 (PubMed, Embase, and UpToDate databases)/inception until 2014	Vegan or lacto-ovo-vegetarian diets/9–96 weeks	Weight	ROB/NR/NR	Low
Massara, 2022 [33]	6/participants with risk factor(s) for diabetes/NR/706	Denmark, Sweden, Iceland, Finland/2008–2020	3 (MEDLINE, Embase, the Cochrane Central Register of Controlled Trials)/inception until 2021	Nordic diet/NR	Weight, BMI, waist circumference, fasting blood glucose, blood pressure, lipid profiles	ROB/GRADE/NCT04094194	Moderate
Termannsen, 2022 [34]	11/participants with T2DM or overweight/48–61/796	USA, Canada, Republic of Korea, New Zealand/1999–2021	4 (MEDLINE, Embase, CINAHL, the Cochrane Central Register of Controlled Trials (CENTRAL))/inception until 2022	Low-fat vegan, low-carbohydrate vegan diet/12–26 weeks	Weight, BMI, fasting blood glucose, blood pressure, lipid profiles	ROB2/GRADE/CRD42021233938	Moderate
Viguiliouk, 2019 [35]	9/participants with T1DM or T2DM/32–61/664	USA, Greece, Brazil, Czech Republic, Korea/NR	3 (MEDLINE, Embase, the Cochrane Central Register of Controlled Trials)/inception until 2018	Vegetarian protein diet, low-fat vegan diet, plant-based protein diet, lacto-vegetarian low-protein diet, low-fat vegan diet, vegetarian diet, low-fat low-glycemic index vegan diet/NR	Weight, BMI, waist circumference, fasting blood glucose, blood pressure, lipid profiles	NR/GRADE/NCT02600377	High

RCT—randomized controlled trial; T2DM—type 2 diabetes mellitus; NR—not reported; ROB—Cochrane risk of bias tool; ^#^ retrieved from the American Dietetic Association’s Evidence Analysis Manual; * mean age or age range.

**Table 2:** Summary of plant-based diets effects on weight, BMI, waist circumference, lipid profiles, fasting blood glucose, and blood pressure.

	Authors, Year	MD/SMD (95% CI)	Sample Size in Number of Studies	*p*-Value	95% Prediction Interval Rule	Small-Study Effects or Excess Significance Bias	*I* ^2^	Credibility of Evidence (Class)
**Weight (kg)**								
	Austin, 2021 [29]	MD = −2.35 (−3.51, −1.19)	N < 1000 (N = 384 in 7 studies)	*p* < 0.001	Including the null value	NR	78.43%	Weak (Class IV)
	Barnard, 2015 [30]	MD = −3.4 (−2.4, −4.4)	N < 1000 (N = 755 in 15 studies)	*p* < 0.001	Including the null value	No small-study effects (Egger’s test: *p* = 0.27)	64.3%	Convincing (Class I)
	Demirci, 2022 [31]	MD = 0.954 (1.515, 0.393)	N < 1000 (N = 934 in 11 studies)	*p* = 0.001	Including the null value	No small-study effects (*p* = 0.425), no publication bias seen	93.75%	Weak (Class IV)
	Huang, 2016 [32]	MD = −2.02 (−1.23, −2.80)	N > 1000 (N = 1151 in 12 studies)	*p* = 0.001	Including the null value	No publication bias seen (Begg’s test *p* = 0.32)	62.3%	Weak (Class IV)
	Massara, 2022 [33]	MD = −2 (−3.24, −0.75)	N < 1000 (N = 706 in 6 studies)	*p* = 0.002	Including the null value	No small-study effects (no publication bias, no Egger’s result)	88%	Weak (Class IV)
	Termannsen, 2022 [34]	MD = −4.1 (−5.9, −2.4)	N < 1000 (N = 697 in 11 studies)	*p* < 0.001	Including the null value	No small-study effects (no publication bias, no Egger’s result)	91%	Weak (Class IV)
	Viguiliouk, 2019 [35]	MD = −2.15 (−2.95, −1.34)	N < 1000 (N = 532 in 9 studies)	*p* < 0.001	Including the null value	No small-study effects (no publication bias, no Egger’s result)	21%	Weak (Class IV)
**BMI (kg/m^2^)**								
	Austin, 2021 [29]	MD = −0.9 (−1.42, −0.38)	N < 1000 (N = 339 in 7 studies)	*p* = 0.001	Including the null value	Statistically significant publication bias (Egger’s test: *p* < 0.005)	85.32%	Weak (Class IV)
	Massara, 2022 [33]	MD = −0.98 (−1.19, −0.77)	N < 1000 (N = 393 in 6 studies)	*p* < 0.001	Including the null value	No small-study effects (no publication bias, no Egger’s result)	19%	Weak (Class IV)
	Termannsen, 2022 [34]	MD = −1.38 (−1.96, −0.8)	N < 1000 (N = 780 in 11 studies)	*p* < 0.001	Including the null value	No small-study effects (no publication bias, no Egger’s result)	89%	Weak (Class IV)
	Viguiliouk, 2019 [35]	MD = −0.74 (−1.09, −0.39)	N < 1000 (N = 614 in 9 studies)	*p* < 0.001)	Including the null value	No small-study effects (no publication bias, no Egger’s result)	60%	Weak (Class IV)
**Waist circumference (cm)**								
	Austin, 2021 [29]	MD = −2.41 (−3.72, −1.09)	N < 1000 (N = 191 in 7 studies)	*p*< 0.001	Including the null value	NR	81.01%	Weak (Class IV)
	Massara, 2022 [33]	MD = −1.32 (−2.2, −0.43)	N < 1000 (N = 454 in 6 studies)	*p* = 0.003	Including the null value	No small-study effects (no publication bias, no Egger’s result)	71%	Weak (Class IV)
	Viguiliouk, 2019 [35]	MD = −2.86 (−3.76, −1.96)	N < 1000 (N = 283 in 9 studies)	*p* < 0.001	Including the null value	No small-study effects (no publication bias, no Egger’s result)	48%	Weak (Class IV)
**LDL-C (mmol/L)**								
	Massara, 2022 [33]	MD = −0.26 (−0.52, 0)	N < 1000 (N = 606 in 6 studies)	*p* = 0.05	Excluding the null value	No small-study effects (no publication bias, no Egger’s result)	89%	Weak (Class IV)
	Termannsen, 2022 [34]	MD = −0.24 (−0.4, −0.07)	N < 1000 (N = 684 in 11 studies)	*p* = 0.005	Including the null value	No small-study effects (no publication bias, no Egger’s result)	58%	Weak (Class IV)
	Viguiliouk, 2019 [35]	MD = −0.12 (−0.2, −0.04)	N < 1000 (N = 602 in 9 studies)	*p* = 0.002	Including the null value	No small-study effects (no publication bias, no Egger’s result)	0%	Weak (Class IV)
**HDL-C (mmol/L)**								
	Massara, 2022 [33]	MD = −0.03 (−0.1, 0.03)	N < 1000 (N = 606 in 6 studies)	*p* = 0.35	Excluding the null value	No small-study effects (no publication bias, no Egger’s result)	75%	Weak (Class IV)
	Termannsen, 2022 [34]	MD = −0.06 (−0.12, 0.01)	N < 1000 (N = 698 in 11 studies)	*p* = 0.08	Excluding the null value	No small-study effects (no publication bias, no Egger’s result)	67%	Weak (Class IV)
	Viguiliouk, 2019 [35]	MD = −0.03 (−0.08, 0.02)	N < 1000 (N = 632 in 9 studies)	*p* = 0.19	Excluding the null value	No small-study effects (no publication bias, no Egger’s result)	66%	Weak (Class IV)
**Triglycerides (mmol/L)**								
	Massara, 2022 [33]	MD = −0.05 (−0.14, 0.05)	N < 1000 (N = 606 in 6 studies)	*p* = 0.34	Excluding the null value	No small-study effects (no publication bias, no Egger’s result)	43%	Weak (Class IV)
	Termannsen, 2022 [34]	MD = 0.11 (−0.08, 0.29)	N < 1000 (N = 698 in 11 studies)	*p* = 0.26	Excluding the null value	No small-study effects (no publication bias, no Egger’s result)	65%	Weak (Class IV)
	Viguiliouk, 2019 [35]	MD = 0.14 (−0.1, 0.38)	N < 1000 (N = 615 in 9 studies)	*p* = 0.26	Excluding the null value	No small-study effects (no publication bias, no Egger’s result)	71%	Weak (Class IV)
**HbA1c (%)**								
	Massara, 2022 [33]	MD = 0.01 (−0.06, 0.08)	N < 1000 (N = 145 in 6 studies)	*p* = 0.79	Excluding the null value	No small-study effects (no publication bias, no Egger’s result)	NR	Weak (Class IV)
	Termannsen, 2022 [34]	MD = −0.18 (−0.29, −0.07)	N < 1000 (N = 687 in 11 studies)	*p* = 0.002	Including the null value	No small-study effects (no publication bias, no Egger’s result)	66%	Weak (Class IV)
	Viguiliouk, 2019 [35]	MD = −0.29 (−0.45, −0.12)	N < 1000 (N = 378 in 9 studies)	*p* < 0.001	Including the null value	No small-study effects (no publication bias, no Egger’s result)	14%	Weak (Class IV)
**Fasting insulin (pmol/L)**								
	Massara, 2022 [33]	MD = −7.83 (−12.26, −3.39)	N < 1000 (N = 393 in 6 studies)	*p* < 0.001	Including the null value	No small-study effects (no publication bias, no Egger’s result)	0%	Weak (Class IV)
	Viguiliouk, 2019 [35]	MD = −7.92 (−27.92, 12.08)	N < 1000 (N = 74 in 9 studies)	*p* = 0.44	Excluding the null value	No small-study effects (no publication bias, no Egger’s result)	NR	Weak (Class IV)
**SBP (mmHg)**								
	Massara, 2022 [33]	MD = −3.55 (−5.12, −1.59)	N < 1000 (N = 533 in 6 studies)	*p* = 0.002	Including the null value	No small-study effects (no publication bias, no Egger’s result)	50%	Weak (Class IV)
	Termannsen, 2022 [34]	MD = 1.28 (−1.54, 4.11)	N < 1000 (N = 466 in 11 studies)	*p* = 0.37	Excluding the null value	No small-study effects (no publication bias, no Egger’s result)	34%	Weak (Class IV)
	Viguiliouk, 2019 [35]	MD = −0.1 (−2.33, 2.52)	N < 1000 (N = 606 in 9 studies)	*p* = 0.94	Excluding the null value	No small-study effects (no publication bias, no Egger’s result)	35%	Weak (Class IV)
**DBP (mmHg)**								
	Massara, 2022 [33]	MD = −1.5 (−2.62, −0.37)	N < 1000 (N = 533 in 6 studies)	*p* = 0.009	Including the null value	No small-study effects (no publication bias, no Egger’s result)	34%	Weak (Class IV)
	Termannsen, 2022 [34]	MD = 0.54 (−1.21, 2.29)	N < 1000 (N = 466 in 11 studies)	*p* = 0.55	Excluding the null value	No small-study effects (no publication bias, no Egger’s result)	37%	Weak (Class IV)
	Viguiliouk, 2019 [35]	MD = 0.53 (−0.5, 1.57)	N < 1000 (N = 606 in 9 studies)	*p* = 0.31	Excluding the null value	No small-study effects (no publication bias, no Egger’s result)	0%	Weak (Class IV)

NR—not reported; BMI—body mass index; HDL-C—high-density lipoprotein; LDL-C—low-density lipoprotein cholesterol; SBP—systolic blood pressure; DBP—diastolic blood pressure; HbA1c—hemoglobin A1c.

## Data Availability

No new data were created or analyzed in this study. Data sharing is not applicable to this article.

## Supplementary Materials

The following supporting information can be downloaded at: https://www.mdpi.com/article/10.3390/nu15102331/s1, Supplementary S1: Preferred Reporting Items for Systematic Reviews and Meta-Analyses (PRISMA) checklist; Supplementary S2: Search strategy for each database; Table S1: AMSTAR 2 assessment of the included systematic reviews; Figure S1: Forest plot comparing the different primary studies’ effect sizes between different plant-based diets on weight (kg); Figure S2: Forest plot comparing the different primary studies’ effect sizes between different plant-based diets on BMI (kg/m^2^); Figure S3: Forest plot comparing the different primary studies’ effect sizes between different plant-based diets on waist circumference (cm); Figure S4: Pooled effect size of plant-based diets on high-density lipoprotein cholesterol (mmol/L) based on findings from the included systematic reviews with meta-analysis; Figure S5: Pooled effect size of plant-based diets on low-density lipoprotein cholesterol (mmol/L) based on findings from the included systematic reviews with meta-analysis; Figure S6: Pooled effect size of plant-based diets on triglyceride cholesterol (mmol/L) based on findings from the included systematic reviews with meta-analysis; Figure S7: Forest plot comparing the different primary studies’ effect sizes between different plant-based diets on triglyceride cholesterol (mmol/L); Figure S8: Funnel plot of the effect estimates on systolic blood pressure, indicating the presence of asymmetry; Figure S9: Funnel plot of the effect estimates on diastolic blood pressure, indicating the presence of asymmetry; Figure S10: Forest plot comparing the different primary studies’ effect sizes between different plant-based diets on HbA1c (mmol/L); Figure S11: Pooled effect size of plant-based diets on blood glucose (mmol/L) based on findings from the included systematic reviews with meta-analysis.
