# Supplementary material for: Effects of Plant-Based Diets on Anthropometric and Cardiometabolic Markers in Adults: An Umbrella Review
Source: Nutrients. 2023 May 16;15(10):2331. doi: 10.3390/nu15102331 (PMC10222061; doi:10.3390/nu15102331)
Supplement: Supplementary file 1 [file nutrients-15-02331-s001.zip › nutrients-2328259-supplementary.pdf]

Supplementary S1: Preferred Reporting Items for Systematic Reviews and Meta-Analyses (PRISMA) checklist

Section and Topic	Item #	Checklist item	Location where item is reported
**TITLE**			
Title	1	Identify the report as a systematic review.	1
**ABSTRACT**			
Abstract	2	See the PRISMA 2020 for Abstracts checklist.	2
**INTRODUCTION**			
Rationale	3	Describe the rationale for the review in the context of existing knowledge.	3-4
Objectives	4	Provide an explicit statement of the objective(s) or question(s) the review addresses.	4
**METHODS**			
Eligibility criteria	5	Specify the inclusion and exclusion criteria for the review and how studies were grouped for the syntheses.	5
Information sources	6	Specify all databases, registers, websites, organisations, reference lists and other sources searched or consulted to identify studies. Specify the date when each source was last searched or consulted.	4-5
Search strategy	7	Present the full search strategies for all databases, registers and websites, including any filters and limits used.	4-5
Selection process	8	Specify the methods used to decide whether a study met the inclusion criteria of the review, including how many reviewers screened each record and each report retrieved, whether they worked independently, and if applicable, details of automation tools used in the process.	5
Data collection process	9	Specify the methods used to collect data from reports, including how many reviewers collected data from each report, whether they worked independently, any processes for obtaining or confirming data from study investigators, and if applicable, details of automation tools used in the process.	6
Data items	10a	List and define all outcomes for which data were sought. Specify whether all results that were compatible with each outcome domain in each study were sought (e.g. for all measures, time points, analyses), and if not, the methods used to decide which results to collect.	6

Section and Topic	Item #	Checklist item	Location where item is reported
	10b	List and define all other variables for which data were sought (e.g. participant and intervention characteristics, funding sources). Describe any assumptions made about any missing or unclear information.	6
Study risk of bias assessment	11	Specify the methods used to assess risk of bias in the included studies, including details of the tool(s) used, how many reviewers assessed each study and whether they worked independently, and if applicable, details of automation tools used in the process.	6
Effect measures	12	Specify for each outcome the effect measure(s) (e.g. risk ratio, mean difference) used in the synthesis or presentation of results.	6-7
Synthesis methods	13a	Describe the processes used to decide which studies were eligible for each synthesis (e.g. tabulating the study intervention characteristics and comparing against the planned groups for each synthesis (item #5)).	6-8
	13b	Describe any methods required to prepare the data for presentation or synthesis, such as handling of missing summary statistics, or data conversions.	7-8
	13c	Describe any methods used to tabulate or visually display results of individual studies and syntheses.	7-8
	13d	Describe any methods used to synthesize results and provide a rationale for the choice(s). If meta-analysis was performed, describe the model(s), method(s) to identify the presence and extent of statistical heterogeneity, and software package(s) used.	7-8
	13e	Describe any methods used to explore possible causes of heterogeneity among study results (e.g. subgroup analysis, meta-regression).	7-8
	13f	Describe any sensitivity analyses conducted to assess robustness of the synthesized results.	7-8
Reporting bias assessment	14	Describe any methods used to assess risk of bias due to missing results in a synthesis (arising from reporting biases).	6-7
Certainty assessment	15	Describe any methods used to assess certainty (or confidence) in the body of evidence for an outcome.	6-7

Section and Topic	Item #	Checklist item	Location where item is reported
**RESULTS**			
Study selection	16a	Describe the results of the search and selection process, from the number of records identified in the search to the number of studies included in the review, ideally using a flow diagram.	8
	16b	Cite studies that might appear to meet the inclusion criteria, but which were excluded, and explain why they were excluded.	NA
Study characteristics	17	Cite each included study and present its characteristics.	Table 1
Risk of bias in studies	18	Present assessments of risk of bias for each included study.	Table 1
Results of individual studies	19	For all outcomes, present, for each study: (a) summary statistics for each group (where appropriate) and (b) an effect estimate and its precision (e.g. confidence/credible interval), ideally using structured tables or plots.	Table 3
Results of syntheses	20a	For each synthesis, briefly summarise the characteristics and risk of bias among contributing studies.	8-9
	20b	Present results of all statistical syntheses conducted. If meta-analysis was done, present for each the summary estimate and its precision (e.g. confidence/credible interval) and measures of statistical heterogeneity. If comparing groups, describe the direction of the effect.	9-10
	20c	Present results of all investigations of possible causes of heterogeneity among study results.	9-10
	20d	Present results of all sensitivity analyses conducted to assess the robustness of the synthesized results.	NA
Reporting biases	21	Present assessments of risk of bias due to missing results (arising from reporting biases) for each synthesis assessed.	8
Certainty of evidence	22	Present assessments of certainty (or confidence) in the body of evidence for each outcome assessed.	Table 2
**DISCUSSION**			
Discussion	23a	Provide a general interpretation of the results in the context of other evidence.	10-14

Section and Topic	Item #	Checklist item	Location where item is reported
	23b	Discuss any limitations of the evidence included in the review.	10-14
	23c	Discuss any limitations of the review processes used.	10-14
	23d	Discuss implications of the results for practice, policy, and future research.	10-14
**OTHER INFORMATION**			
Registration and protocol	24a	Provide registration information for the review, including register name and registration number, or state that the review was not registered.	4
	24b	Indicate where the review protocol can be accessed, or state that a protocol was not prepared.	4
	24c	Describe and explain any amendments to information provided at registration or in the protocol.	NA
Support	25	Describe sources of financial or non-financial support for the review, and the role of the funders or sponsors in the review.	14
Competing interests	26	Declare any competing interests of review authors.	15
Availability of data, code and other materials	27	Report which of the following are publicly available and where they can be found: template data collection forms; data extracted from included studies; data used for all analyses; analytic code; any other materials used in the review.	NA

From: Page MJ, McKenzie JE, Bossuyt PM, Boutron I, Hoffmann TC, Mulrow CD, et al. The PRISMA 2020 statement: an updated guideline for reporting systematic reviews. BMJ 2021;372:n71. doi: 10.1136/bmj.n71

For more information, visit: http://www.prisma-statement.org/

Supplementary S2: Search strategy for each database.

Database	Index and keyword terms	Results
PubMed	(((plant-based diet*[Title/Abstract]) OR (plant based diet*[Title/Abstract]) OR (plant-based nutrition[Title/Abstract]) OR (plant based nutrition[Title/Abstract]) OR (plant-based food*[Title/Abstract]) OR (plant based food*[Title/Abstract]) OR (vegetarian*[Title/Abstract]) OR (vegan[Title/Abstract]) OR lacto-vegetarian*[Title/Abstract] OR lacto vegetarian*[Title/Abstract] OR lacto-ovo vegetarian*[Title/Abstract] OR lacto ovo vegetarian*[Title/Abstract] OR plant food[Title/Abstract]) AND (Overweight[Title/Abstract] OR obesity[Title/Abstract] OR "body weight"[Title/Abstract] OR "weight loss"[Title/Abstract] OR "weight gain"[Title/Abstract] OR adiposity[Title/Abstract] OR "body mass index"[Title/Abstract])) AND ("systematic review"[Title/Abstract] OR "meta-analysis"[Title/Abstract])	42
EMBASE	('plant-based diet*':ab,ti OR 'plant based diet*':ab,ti OR 'plant-based nutrition':ab,ti OR 'plant based nutrition':ab,ti OR 'plant-based food*':ab,ti OR 'plant based food*':ab,ti OR vegetarian*:ab,ti OR 'lacto vegetarian*':ab,ti OR 'lacto-ovo vegetarian*':ab,ti OR 'lacto ovo vegetarian*':ab,ti OR 'plant food':ab,ti) AND (overweight:ab,ti OR obesity:ab,ti OR 'body weight':ab,ti OR 'weight loss':ab,ti OR 'weight gain':ab,ti OR adiposity:ab,ti OR 'body mass index':ab,ti) AND ('systematic review':ab,ti OR 'meta-analysis':ab,ti)	68
The Cochrane Library (reviews only)	(plant-based diet*) OR (plant based diet*) OR (plant-based nutrition) OR (plant based nutrition) OR (plant-based food*) OR (plant based food*) OR (vegetarian*) OR (vegan) OR lacto-vegetarian* OR lacto vegetarian* OR lacto-ovo vegetarian* OR lacto ovo vegetarian* OR plant food in Title Abstract Keyword AND overweight OR obesity OR "body weight" OR "weight loss" OR "weight gain" OR adiposity OR "body mass index" in Title Abstract Keyword - (Word variations have been searched)	5
CINAHL	AB ((plant-based diet*) OR (plant based diet*) OR (plant-based nutrition) OR (plant based nutrition) OR (plant-based food*) OR (plant based food*) OR (vegetarian*) OR vegan OR lacto-vegetarian* OR lacto vegetarian* OR lacto-ovo vegetarian* OR lacto ovo vegetarian* OR plant food) AND AB (overweight OR obesity OR "body weight" OR "weight loss" OR "weight gain" OR adiposity OR "body mass index") AND AB ("systematic review" OR "meta-analysis")	30
Scopus	(TITLE-ABS-KEY ((plant-based AND diet*) OR (plant AND based AND diet*) OR (plant-based AND	75

	nutrition) OR (plant AND based AND nutrition) OR (plant-based AND food*) OR (plant AND based AND food*) OR (vegetarian*) OR lacto-vegetarian* OR lacto AND vegetarian* OR lacto-ovo AND vegetarian* OR lacto AND ovo AND vegetarian* OR vegan OR plant AND food) AND ALL (overweight OR obesity OR "body weight" OR "weight loss" OR "weight gain" OR adiposity OR "body mass index") AND ALL ("systematic review" OR "meta-analysis"))	
Web of Science	(plant-based diet*) OR (plant based diet*) OR (plant-based nutrition) OR (plant based nutrition) OR (plant-based food*) OR (plant based food*) OR (vegetarian*) OR lacto-vegetarian* OR lacto vegetarian* OR lacto-ovo vegetarian* OR lacto ovo vegetarian* OR vegan OR plant food (Topic) and overweight OR obesity OR “body weight” OR “weight loss” OR “weight gain” OR adiposity OR “body mass index” (Topic) and “systematic review” OR “meta-analysis” (Topic)	134

Table S1: AMSTAR2 assessment of the included systematic reviews

**Author, Year**	**Q1**	**Q2***	**Q3**	**Q4***	**Q5**	**Q6**	**Q7***	**Q8**	**Q9*[a]**	**Q9[b]**	**Q10**	**Q11*[a]**	**Q11[b]**	**Q12***	**Q13**	**Q14**	**Q15***	**Q16**	**Overall**
Austin et al., 2021	N	PY	N	PY	Y	Y	N	PY	Y	-	Y	Y	-	Y	Y	Y	Y	Y	Moderate
Barnard et al., 2015	N	Y	Y	PY	Y	Y	N	Y	PY	-	Y	Y	-	Y	Y	Y	Y	Y	Moderate
Demirci et al., 2022	N	Y	Y	PY	N	N	N	Y	N	N	N	Y	Y	N	N	Y	Y	N	Low
Huang et al., 2016	N	N	Y	Y	Y	N	N	Y	Y	-	N	Y	-	Y	Y	Y	Y	Y	Low
Massara et al., 2022	Y	Y	N	Y	Y	N	Y	Y	N	Y	Y	Y	-	Y	N	Y	Y	Y	Moderate
Termannsen et al., 2022	Y	Y	N	Y	Y	N	Y	Y	Y	-	Y	Y	-	Y	Y	Y	Y	Y	Moderate
Viguiliouk et al., 2018	Y	Y	Y	PY	Y	Y	N	Y	Y	-	Y	Y	-	Y	Y	Y	N	Y	High

N = No, Y = Yes, PY = Partially Yes, * = critical domains

Q1: Did the research questions and inclusion criteria for the review include the components of PICO?
Q2: Did the report of the review contain an explicit statement that the review methods were established prior to conduct of the review and did the report justify any significant deviations from the protocol?
Q3: Did the review authors explain their selection of the study designs for inclusion in the review?
Q4: Did the review authors use a comprehensive literature search strategy?
Q5: Did the review authors perform study selection in duplicate?
Q6: Did the review authors perform data extraction in duplicate?
Q7: Did the review authors provide a list of excluded studies and justify the exclusions?
Q8: Did the review authors describe the included studies in adequate detail?
Q9: Did the review authors use a satisfactory technique for assessing the risk of bias (RoB) in individual studies that were included in the review?
Q10: Did the review authors report on the sources of funding for the studies included in the review?
Q11: If meta-analysis was justified did the review authors use appropriate methods for statistical combination of results?
Q12: If meta-analysis was performed did the review authors assess the potential impact of RoB in individual studies on the results of the meta-analysis or other evidence synthesis?
Q13: Did the review authors account for RoB in individual studies when interpreting/discussing the results of the review?
Q14: Did the review authors provide a satisfactory explanation for, and discussion of, any heterogeneity observed in the results of the review?
Q15: If they performed quantitative synthesis did the review authors carry out an adequate investigation of publication bias (small study bias) and discuss its likely impact on the results of the review?
Q16: Did the review authors report any potential sources of conflict of interest, including any funding they received for conducting the revie

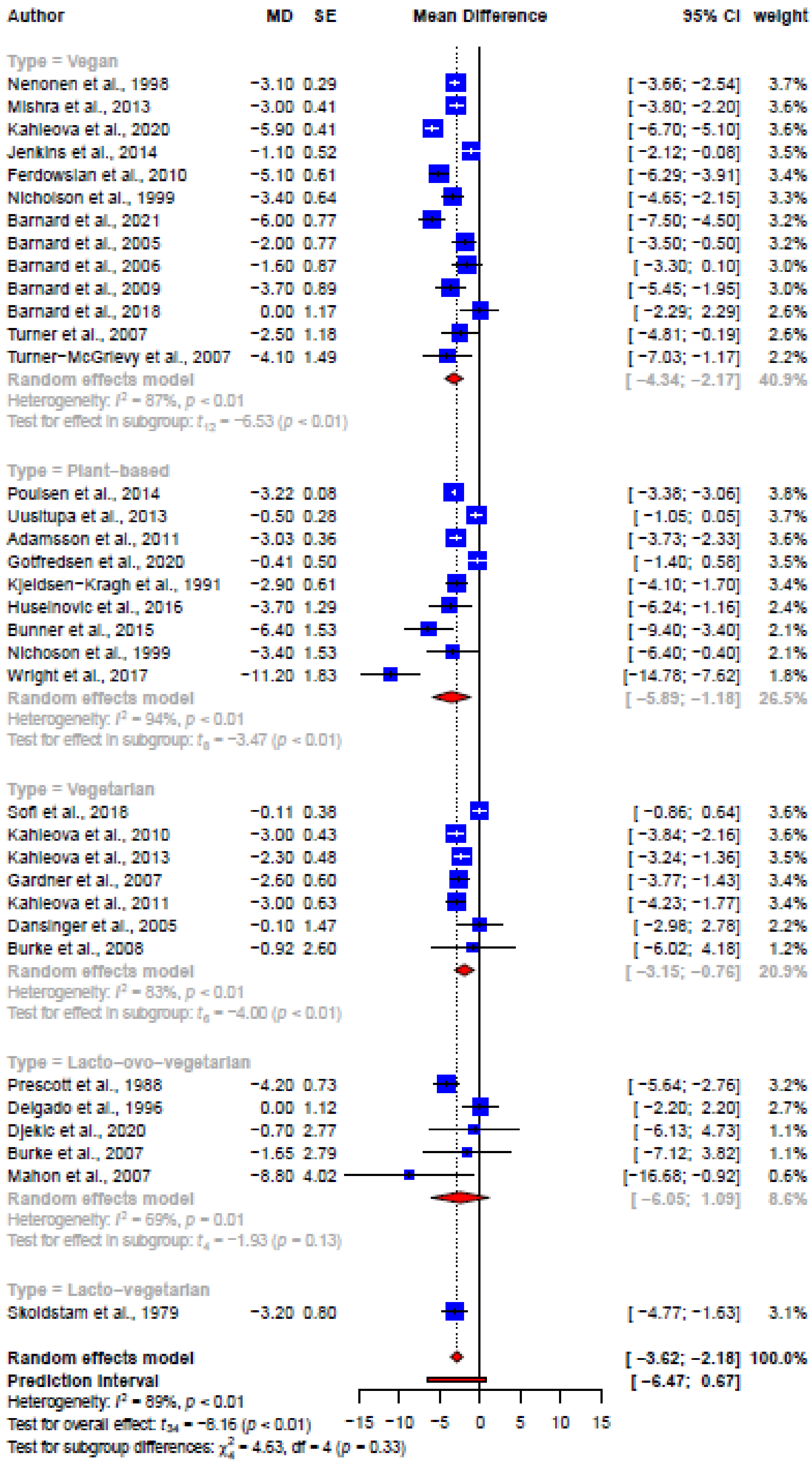

Figure S1: Forest plot comparing the different primary studies' effect sizes between different plant-based diets on weight (kg).

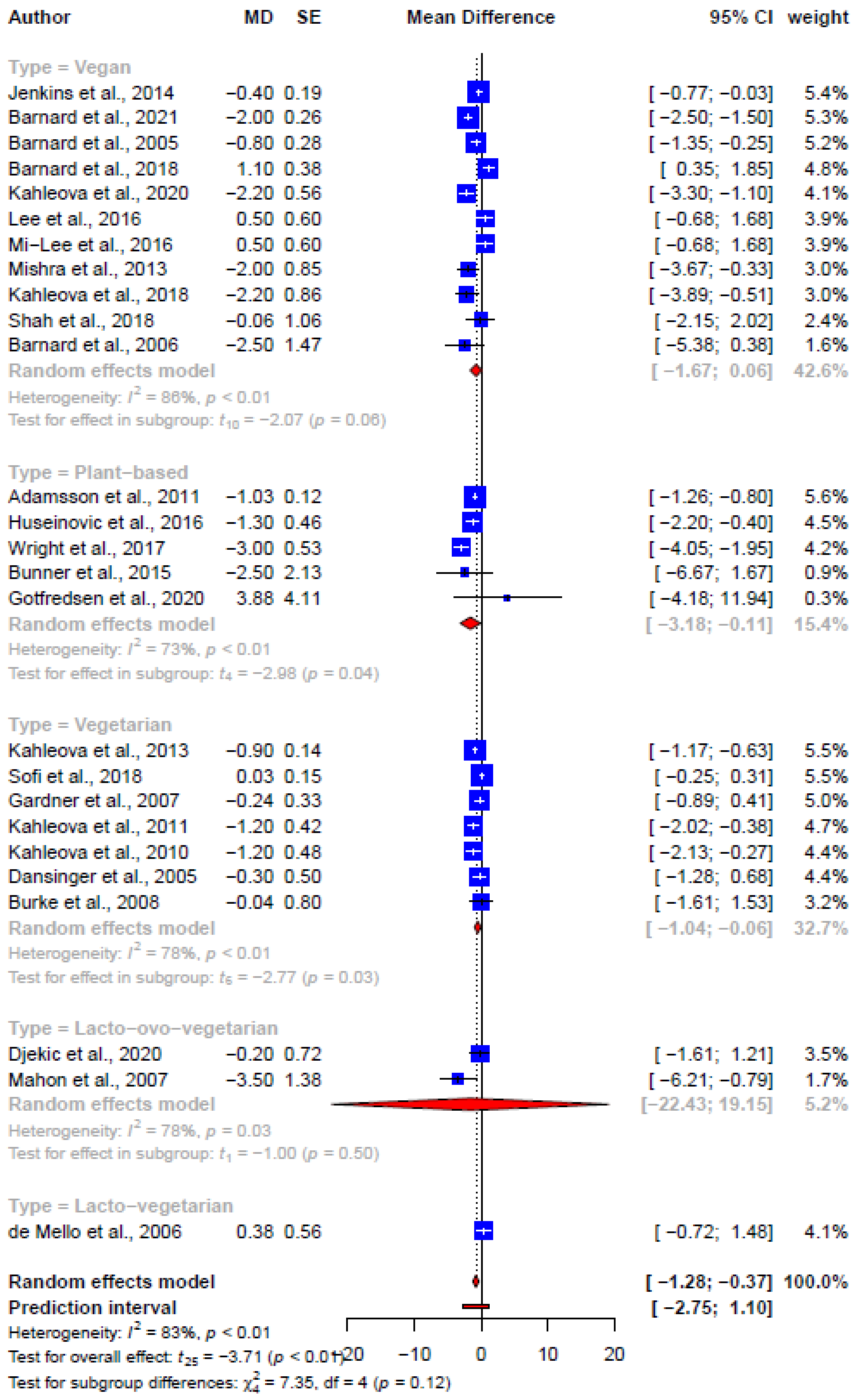

Figure S2: Forest plot comparing the different primary studies' effect sizes between different plant-based diets on BMI (kg/m^2).

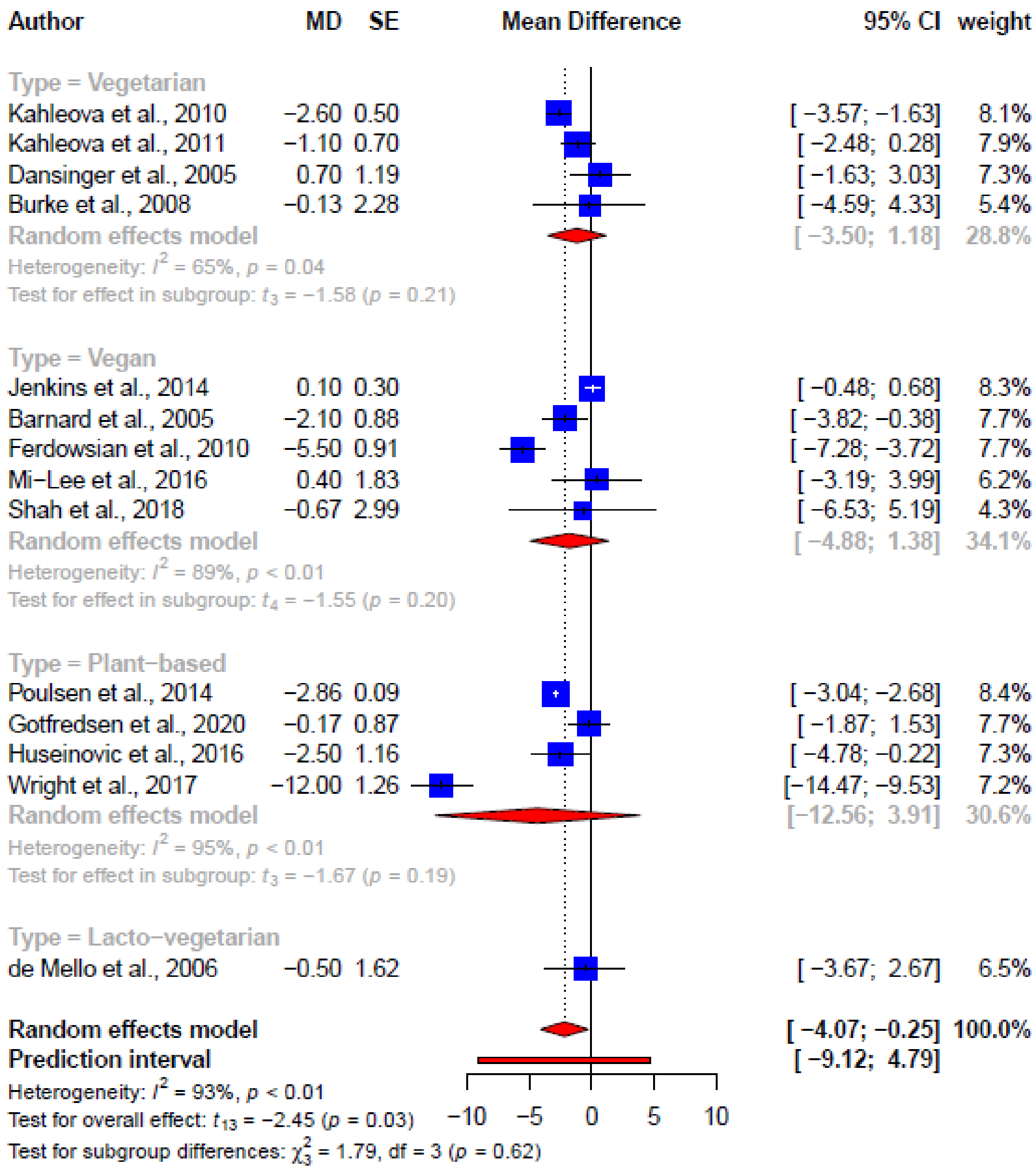

Figure S3: Forest plot comparing the different primary studies' effect sizes between different plant-based diets on waist circumference (cm).

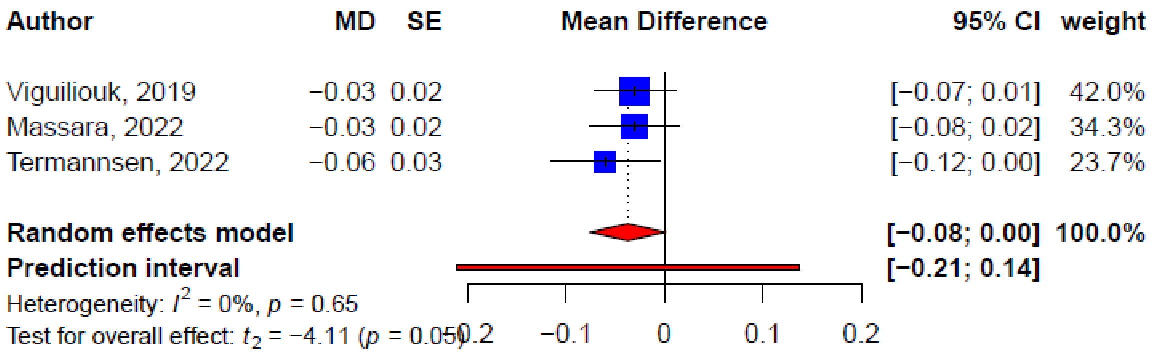

Figure S4: Pooled effect size of plant-based diets on high-density lipoprotein cholesterol (mmol/L) based on findings from the included systematic reviews with meta-analysis.

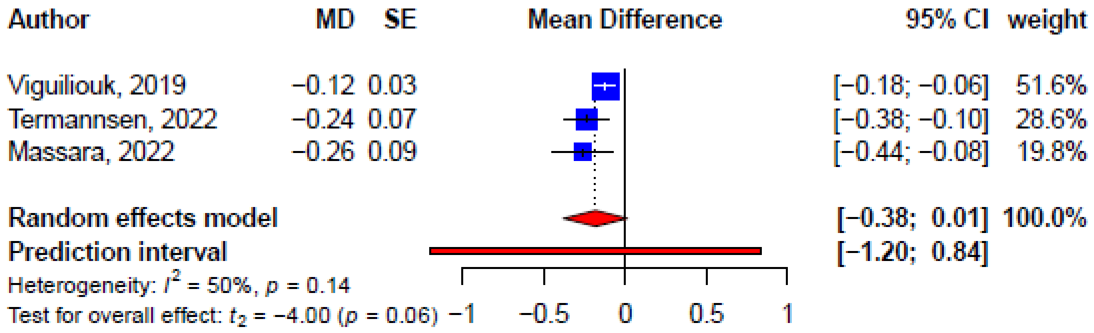

Figure S5: Pooled effect size of plant-based diets on low-density lipoprotein cholesterol (mmol/L) based on findings from the included systematic reviews with meta-analysis.

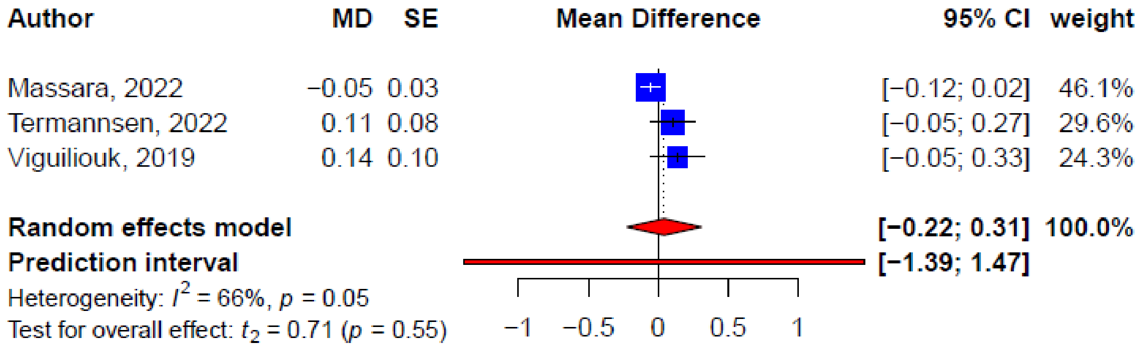

Figure S6: Pooled effect size of plant-based diets on triglyceride cholesterol (mmol/L) based on findings from the included systematic reviews with meta-analysis.

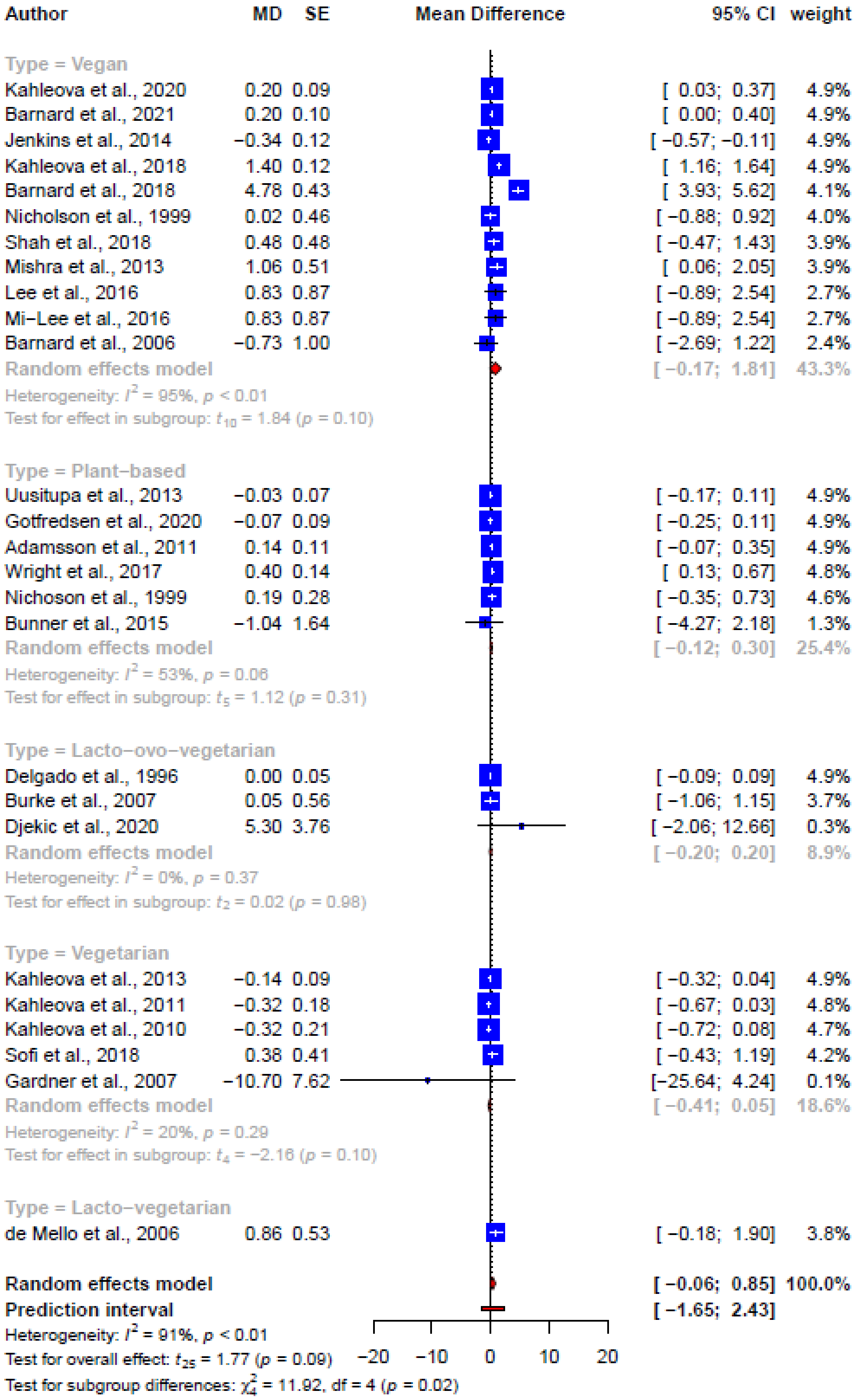

Figure S7: Forest plot comparing the different primary studies' effect sizes between different plant-based diets on triglyceride cholesterol (mmol/L).

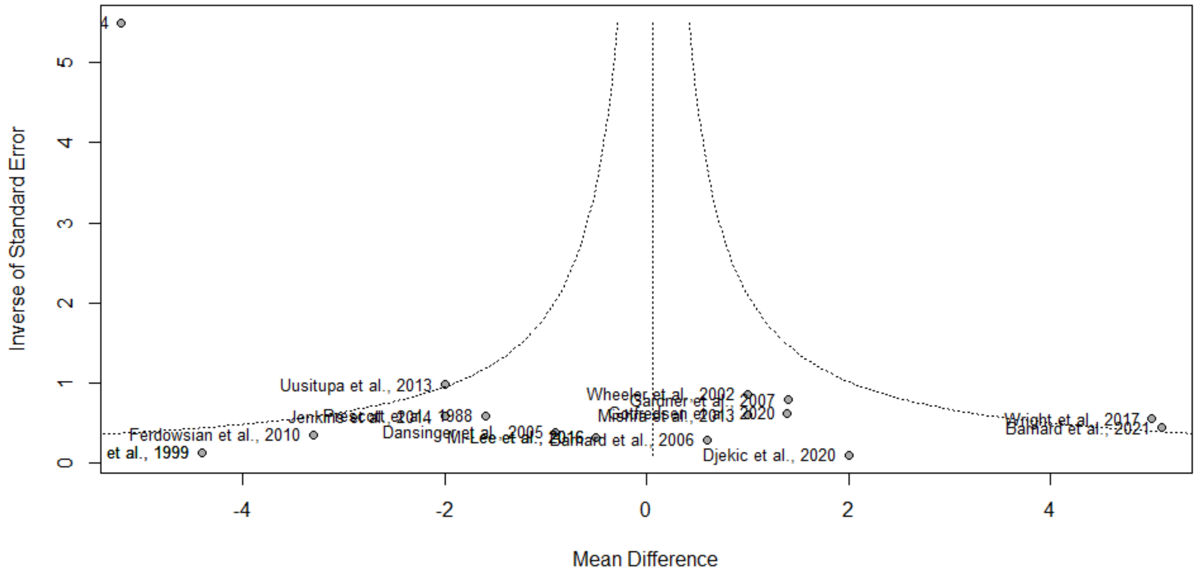

Figure S8: Funnel plot of the effect estimates on systolic blood pressure, indicating the presence of asymmetry.

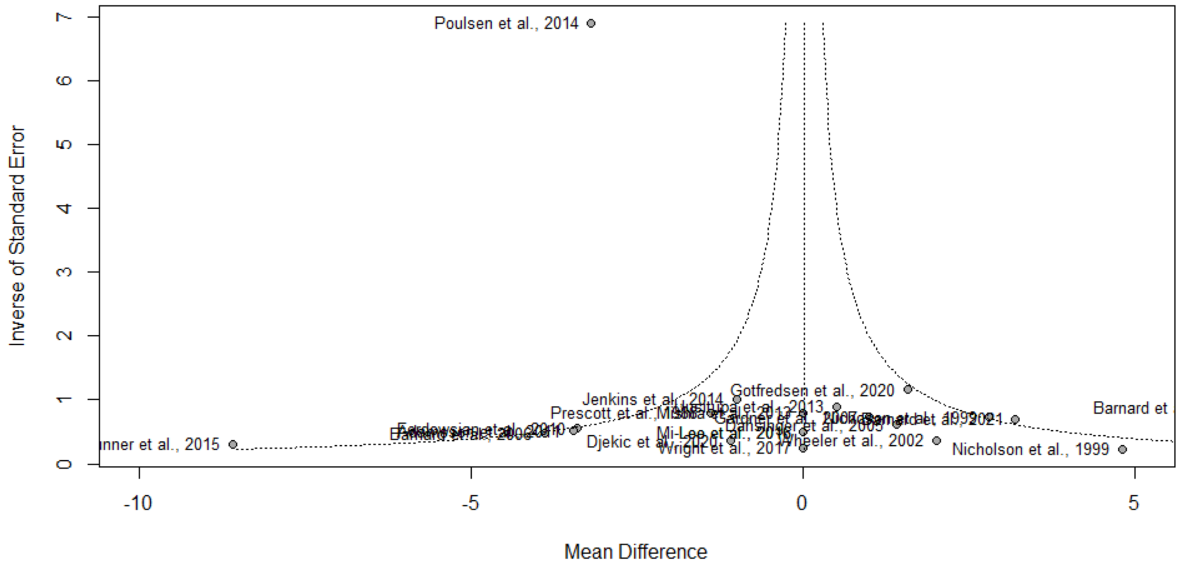

Figure S9: Funnel plot of the effect estimates on diastolic blood pressure, indicating the presence of asymmetry.

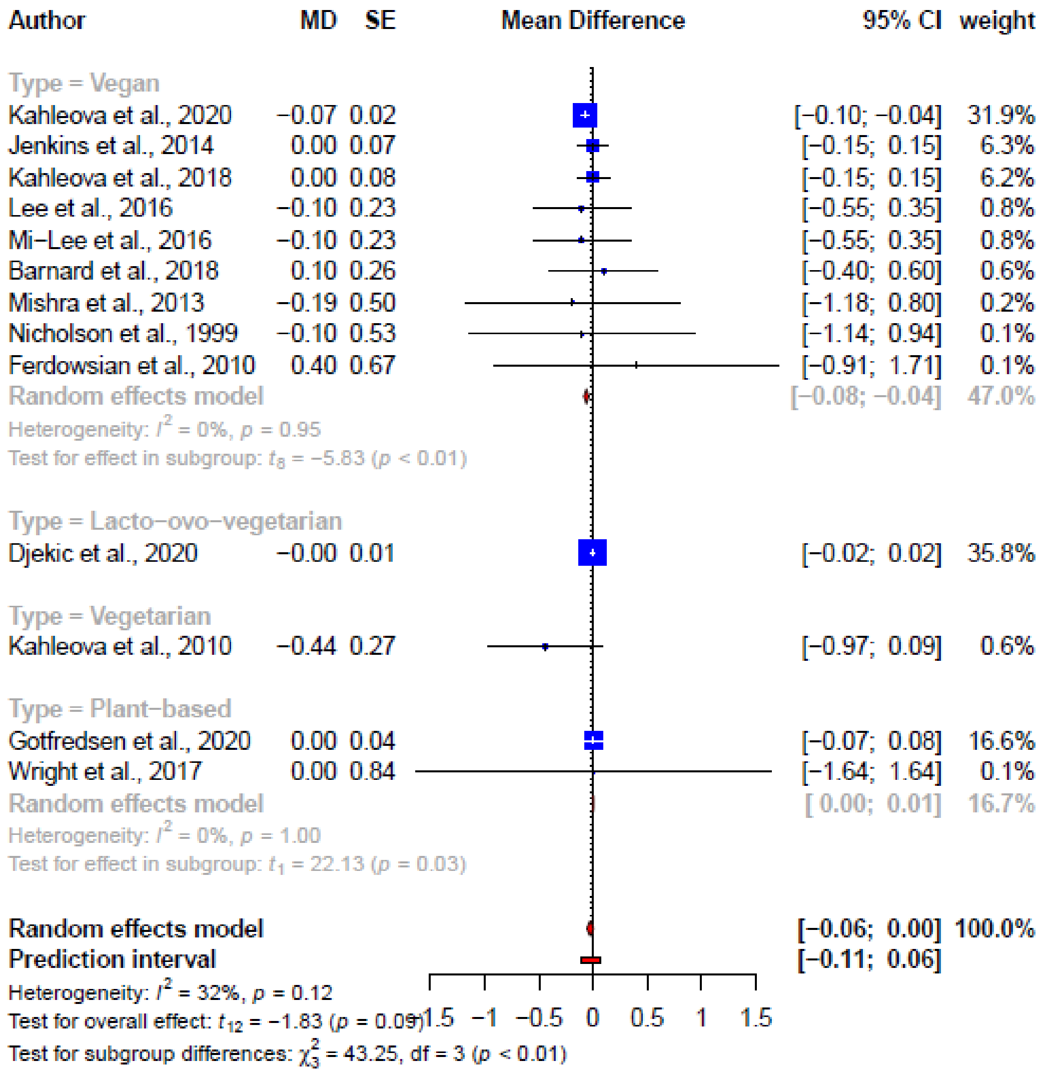

Figure S10: Forest plot comparing the different primary studies' effect sizes between different plant-based diets on HbA1c (mmol/L).

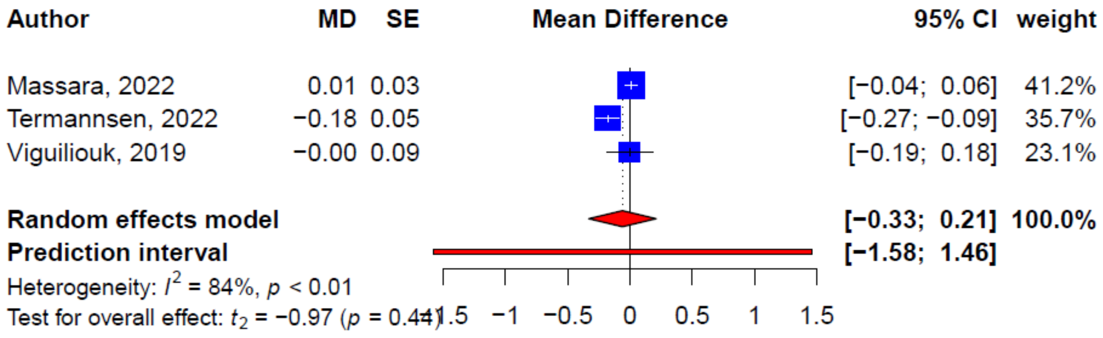

Figure S11: Pooled effect size of plant-based diets on blood glucose (mmol/L) based on findings from the included systematic reviews with meta-analysis.
